# Reversing Epigenetic Gene Silencing to Overcome Immune Evasion in CNS Malignancies

**DOI:** 10.3389/fonc.2021.719091

**Published:** 2021-07-15

**Authors:** Nivedita M. Ratnam, Heather M. Sonnemann, Stephen C. Frederico, Huanwen Chen, Marsha-Kay N. D. Hutchinson, Tyrone Dowdy, Caitlin M. Reid, Jinkyu Jung, Wei Zhang, Hua Song, Meili Zhang, Dionne Davis, Mioara Larion, Amber J. Giles, Mark R. Gilbert

**Affiliations:** Neuro-Oncology Branch, National Cancer Institute, Bethesda, MD, United States

**Keywords:** immunotherapy, CNS malignancies, GSK126, immune evasion, epigenetic gene silencing

## Abstract

Glioblastoma (GBM) is an aggressive brain malignancy with a dismal prognosis. With emerging evidence to disprove brain-immune privilege, there has been much interest in examining immunotherapy strategies to treat central nervous system (CNS) cancers. Unfortunately, the limited success of clinical studies investigating immunotherapy regimens, has led to questions about the suitability of immunotherapy for these cancers. Inadequate inherent populations of tumor infiltrating lymphocytes (TILs) and limited trafficking of systemic, circulating T cells into the CNS likely contribute to the poor response to immunotherapy. This paucity of TILs is in concert with the finding of epigenetic silencing of genes that promote immune cell movement (chemotaxis) to the tumor. In this study we evaluated the ability of GSK126, a blood-brain barrier (BBB) permeable small molecule inhibitor of EZH2, to reverse GBM immune evasion by epigenetic suppression of T cell chemotaxis. We also evaluated the *in vivo* efficacy of this drug in combination with anti-PD-1 treatment on tumor growth, survival and T cell infiltration in syngeneic mouse models. GSK126 reversed H3K27me^3^ in murine and human GBM cell lines. When combined with anti-PD-1 treatment, a significant increase in activated T cell infiltration into the tumor was observed. This resulted in decreased tumor growth and enhanced survival both in sub-cutaneous and intracranial tumors of immunocompetent, syngeneic murine models of GBM. Additionally, a significant increase in CXCR3^+^ T cells was also seen in the draining lymph nodes, suggesting their readiness to migrate to the tumor. Closer examination of the mechanism of action of GSK126 revealed its ability to promote the expression of IFN-γ driven chemokines CXCL9 and CXCL10 from the tumor cells, that work to traffic T cells without directly affecting T maturation and/or proliferation. The loss of survival benefit either with single agent or combination in immunocompromised SCID mice, suggest that the therapeutic efficacy of GSK126 in GBM is primarily driven by lymphocytes. Taken together, our data suggests that in glioblastoma, epigenetic modulation using GSK126 could improve current immunotherapy strategies by reversing the epigenetic changes that enable immune cell evasion leading to enhanced immune cell trafficking to the tumor.

## Introduction

Glioblastoma (GBM) accounts for approximately half of all primary malignant brain tumors in the United States. It is one of the most aggressive malignancies with a five-year survival of less than 10% (1). Standard of care for patients with GBM, currently surgical resection followed by chemo-radiation, has only shown a modest impact on survival (2). At the cellular level, GBM is characterized by both inter- and intra-tumoral heterogeneity, making it hard to successfully apply conventional therapeutics, particularly molecular targeted strategies (3)

Historically, the brain was considered to be immune privileged, separated from the circulatory immune environment by the BBB and the blood-cerebrospinal fluid barrier (BCSFB) (4). However, studies of spontaneous infections, aging and autoimmune disorders like multiple sclerosis and Alzheimer’s disease have shown the presence of surveilling lymphocytes, specifically T cells in the brain, proving that under diseased condition immune cells are able to access the brain despite the physical barriers (4, 5). In addition, recent studies have also pointed out to the existence of tissue resident-memory T cells in the healthy human brain with a primary immune surveillance function alongside a prominent role in the development of fetal to adult brain together with microglia (6, 7). These findings coupled with the re-discovery of brain lymphatics provides evidence that tumor antigens could circulate to the cervical lymph nodes, where they would be able to interact with immune cells has provided rationale for the use of immunotherapy to treat patients with GBM (8). There are growing numbers of clinical studies being performed to evaluate several immunotherapy strategies such as use of immune checkpoint inhibitors (ICIs), peptide vaccines, engineered T cells such as CAR T cells, replication competent viral therapy and others (9). However, despite testing in a large series of clinical trials, there has been no confirmation of efficacy of these approaches (9).

GBMs are considered to be “cold” tumors with a sparse infiltration of lymphocytes, specifically T cells (TILs). In comparison to many other solid tumors, gliomas have a much lower tumor mutational burden and hence lower neoantigen expression, making them far less immunogenic. Studies by Ridley and Cavanaugh have demonstrated that 31% of the analyzed patient tumors had definite T cell infiltration, most of which were perivascular and diffuse, 29% patients had “slight infiltration” and 41% patients had no T cell infiltration at all (10). Studies examing the phenotype of TILs have also shown that glioma TILs have a high expression of PD-1 and Tim-3 which are markers of exhaustion (11). The local immunosuppressive environment characterized by elevated levels of cytokines like IL-10, TGFβ and IDO together with large numbers of PD-L1 expressing monocytes contribute to the exhaustive phenotype of the TILs (12–14). Yet, positive correlations have been established between lymphocyte infiltration and survival in GBM patients (15).

Increasing the trafficking of lymphocytes to the tumor may improve the efficacy of immunotherapy. However, in order for immunotherapy to be successful, not only do immune cells need to navigate the BBB and BCSFB but also have to survive the suppressive tumor micro-environment. Under these circumstances, it is essential to increase the number of cytotoxic lymphocytes entering the brain, to give the immunotherapeutic strategy an opportunity to mount a therapeutic and potentially durable response.

According to the theory of “immune surveillance”, the immune system has the ability to eliminate developing tumor cells in the early stages of tumor initiation (16). However, as tumorigenesis progresses, “immune edited” tumors devise several mechanisms to escape immune attack (16). One of the ways in which tumor cells circumvent immune cells is by epigenetically silencing the expression of chemoattractant cytokines. In the present study we investigated the ability of a small molecule inhibitor GSK126 to inhibit EZH2, a histone methyltransferase, to reverse the silencing of these chemokines. EZH2 is a part of the Polycomb Repressor Complex 2 (PRC2) that is involved in transcriptional repression of genes by catalyzing the transfer of methyl group on histone H3 at lysine 27(H3K27) (17). Studies conducted in other solid tumor models like melanoma, ovarian and prostate cancer have demonstrated the ability of GSK126 to increase tumor T cell infiltration leading to decreased tumor growth in murine models of these diseases (17–19). In this study we examined the clinical relevance of GSK126 in murine models of glioblastoma.

## Materials and Methods

### Tumor Cell Culture, Treatment, and Preparation of Conditioned Medium

Human GBM cell lines used include A172 and U251 which were cultured in Dulbecco’s Modified Minimum Essential Medium (DMEM) with 10% Fetal Bovine Serum (FBS) and 1x Penicillin-Streptomycin Glutamate (PSG). Murine cell lines GL261 and CT2A firefly luciferase -mCherry were cultured in DMEM with 10%FBS and 1%PSG. For collecting conditioned media, human cells were cultured in and treated with 500nM GSK126 (Selleckchem; S706) dissolved in DMSO and/or 10ng/mL recombinant IFNγ (human) (Peprotech) in complete medium for 24h following which they were washed with PBS and incubated for another 24h in serum free RPMI1640 medium 1% PSG, 1% MEM non-essential amino acids solution, 15mM HEPES, 1mM sodium pyruvate and 55μM 2-mercaptoethanol for all transwell migration assays. Once collected, conditioned medium was centrifuged, filtered and aliquoted into 1mL aliquots stored at -80°C. For murine cells, they were pre-treated with 500nM GSK126 for 48h prior to treatment with 10ng/mL recombinant IFNγ for 24h following which they were incubated for 24h in serum-free RPMI as mentioned above. The condition medium was collected and processed similarly. For RT-PCR experiments, human cells were treated and cultured with 500nM GSK126 and/or 10ng/mL IFNγ for 24h in DMEM with 10%FBS and 1%PSG. Mouse cells were pre-treated with 500nM GSK126 for 48h and then treated with 10ng/mL IFNγ for an additional 24h in DMEM with 10%FBS and 1%PSG.

### RT-PCR

Total RNA was extracted using TRIzol reagent (Invitrogen). Reverse transcription was performed using with Superscript III first strand synthesis system to obtain cDNA. RT-PCR was performed using the generated cDNA on an ABI Quant Studio7 and analyzed. Gene expression was normalized to GAPDH.

### Transwell Migration Assays

For transwell migration assays, 24mm, 0.4µM transwell polycarbonate inserts from Sigma Aldrich (CLS3412) were used in 24 well plates. 650µL of thawed CM was added to the bottom chamber and 5x10^5^ T cells in 100µL of serum free RPMI1640 medium with 1%PSG, 1% MEM non-essential amino acids solution, 15mM HEPES, 1 mM sodium pyruvate and 55μM 2-mercaptoethanol was added into each transwell insert. A blank well with medium alone, an input only well and positive control wells with medium containing recombinant CXCL9 and CXCL10 were also maintained during the course of each experiment. The assay was incubated for 6h following which a 100µL of medium from the bottom chamber was mixed well with 100µL of equilibrated CellTitre-Glo reagent in a luminometer plate. Subsequently, the plate was incubated for 30 minutes in the dark and luminescence read on a Polar Star Optima (BGM Labtech) luminometer.

### Isolation and Activation of Murine T Cells

Spleens were harvested from C57BL/6 (albino) mice at appx.6-8 weeks of age. T cells were negatively selected using the Magnisort mouse T cell enrichment kit (Invitrogen). The T cells were activated using 96-well flat bottom plate coated with 1mg/mL of anti-CD3 (Biolegend,17A2) antibody. The T cells were then plated using RPMI1640 medium with 10%FBS,1% PSG, 1% MEM non-essential amino acids solution, 15mM HEPES, 1mM sodium pyruvate and 55μM 2-mercaptoethanol to which soluble 1mg/mL of anti-CD28 (Biolegend, 37.51) antibody was added. The cells were then incubated for three days.

### Animal Studies

All animal experiments were performed following the guidelines stipulated by the NCI-Bethesda Animal Care and Use Committee. All murine studies were performed using female albino C57BL/6 mice, 6-8 weeks of age, procured from Jackson Laboratories (Bar Harbor, ME). For sub-cutaneous tumor studies, 6x10^6^ cells of stably transduced CT2A glioma cells with mCherry-firefly luciferase were injected in 100µL of PBS. For intracranial tumor studies, 1x10^3^ CT2A cells with mCherry- firefly luciferase was injected in 2µL PBS. GSK126 for *in vivo* studies was obtained from the NCI- Drug Synthesis and Chemistry Branch and dissolved in 20% SBE-β-Cyclodextrin (MedChemExpress, HY-17031) pH 4-4.5 with 1N acetic acid. Vehicle was 20% SBE-β-Cyclodextrin pH 4-4.5 with 1N acetic acid. Water-soluble dexamethasone (Sigma Aldrich; D2915) was administered at 1mg/kg/day also by intraperitoneal injection. Anti PD-1(*InVivo*MAb; BE0146) or isotype control, rat IgG2a (*InVivo*MAb; BE0089) were also injected intraperitoneally. Subcutaneous tumor growth was measured using calipers and thereafter tumor volume was calculated using the formula for the volume of an ellipsoid given below.

Volume=((longer diameter×(shorter  diameter 2))×(π)/6)

In the case of intracranial tumors, tumor growth was measured using the luminescence reader IVIS Ilumina and analyzed using LivingImage Software.

### Immunofluorescence Analysis

H3K27me^3^ (C36B11) and EZH2 (D2C9) antibodies were purchased from Cell Signaling Technology. Tumor tissues harvested from mice were frozen in OCT. Frozen tumor tissue was fixed in ice-cold methanol and blocked in 5% goat serum in PBS after permeabilization with 0.5%Triton-X. They were then stained at 4°C overnight with primary antibody. Subsequently tissues were washed and stained with secondary antibody goat anti-rabbit IgG (H+L), Alexa flour 488 (Thermo Fisher Scientific; A1108) at room temperature for 1h. Finally, the tissues were stained with 1x DAPI (Molecular Probes/Invitrogen; D3571) washed and mounted using fluorescent mounting agent (DAKO; S3023). For cellular staining, 8-chamber slides were used and coated with 0.1mg/mL Poly-L-lysine. 1x10^5^ cells were cultured for 24-48h and then treated with GSK126 and/or IFNγ for 24h. Cell were then washed in PBS and fixed using 4% paraformaldehyde. Permeabilization, blocking and staining with primary and secondary antibodies were carried out as described above. Once stained the cells were mounted with Vectasheild with DAPI (Vector Laboratories; H1200). All stained tissues and cells were imaged on a Zeiss LSM 880 or Zeiss LSM 780 confocal microscopes. The images were analyzed and quantified using ImageJ software.

### Flow Cytometry Analysis

αCD45-A488 (30F-11), αCD62L-BV785 (MEL-14), αCXCR3-BV510 (CXCR3-173) and αCD69-PE/Cy5 (H1.2F3) were purchased from Biolegend. αCD3-BV605 (17A2), αCD4-BUV496 (53-6.7), αCD8-BUV805 (PC61), αCD25-BV650 (PC61), αCD44- BB700 (IM7), αPD-1-BUV395(J43), αNK1.1-BV711 (PK136), αIFNγ-BUV737(XMG1.2) and αFoxP3-BV421 (MF23) were purchased from Becton Dickson. αKi67-PE (SolA15) was procured from eBioscience and LIVE/DEAD fixable blue dead cell staining kit (L34961) was purchased from Thermo Fisher Scientific. All cells were stained at room temperature in FACS buffer (1% BSA and 0.01% sodium azide in PBS) for 30mins and then fixed with 4% paraformaldehyde. The stained cells were resuspended in FACS buffer and run-on BD LSR Fortessa X-50 machines and analyzed using the Flowjo software, version 9.9.6. and version 10.6.1.

### Liquid Chromatography-Mass Spectrometry Sample Preparation

Samples were collected from mice with three biological replicates. Serum (50 µL) was transferred to 200 μL ice-chilled (4°C) MilliQ H_2_O. Tissue (~16 mg) was measured from subcutaneous and intracranial samples followed by addition of 250 μL MilliQ H_2_O. Then, samples were sonicated at 40 amps (~30 s) until homogeneous. 80 μL of at 0.150 µg/mL debrisoquine in 60% methanol (MeOH)/40% water(aq) reagent was added. 500 μL chilled (-20°C) methanol was added, vortexed (med) and incubated 15 min on ice. 250 μL chilled (-20°C) Chloroform was added, vortexed (high) and incubated 20 min in ice on rotating mixer. Mixture was centrifuged (13,000x g) for 18 min at 4°C. 705 μL of hydrophilic upper layer was aspirated and transferred to separate 1.5 mL microtubes, dried to completion under N2 gas sample concentrator, and stored at -80°C until LC/MS quantification of GSK126. This data is available at the NIH Common Fund’s National Metabolomics Data Repository (NMDR) website, the Metabolomics Workbench, https://www.metabolomicsworkbench.org, where it has been assigned Project ID PR001160. The data can be accessed directly *via* it’s Project DOI: 10.21228/M8RT34 This work is supported by NIH grant, U2C- DK119886.

### LC-MS/MS Quantitative Analysis

Prior to LC/MS analysis, samples were resuspended in 60 MeOH (aq) at 80 µL prior to LC injection.

LC-MS/MS measurement of GSK126 was achieved *via* Agilent 6545 quadrupole time-of-flight mass spectrometer coupled with ultra-high-pressure liquid chromatography (Q-TOF UHPLC/MS) on the 1290 Infinity II system. Using Masshunter Qtof Quant-My-Way 10.0 software, GSK126 was detected at elution time 2.6 min using precursor ion m/z 527.3129 and transition m/z 375.2183 generated *via* N_2_ gas collision-induced fragmentation (CID) at a collision energy (CE) of 12 V. Internal standard (IS) debrisoquine detected at elution time of 2.5 mins with precursor m/z 176.1182 and transition m/z 134.0964 generated at CE 12V. Internal standard 0.150 µg/mL debrisoquine (IS) was added to each calibration standard preparation (consisting of 0, 0.150, 0.25, 0.50, 0.75, 1.0, 5.0, 7.5, 10 µg/mL GSK 126) as well as each sample in order to conduct qualitative signal correction. For calibration curve, two technical replicates were injected (6 μL) per standard. Continuous accurate mass correction was achieved by infusing proprietary Agilent Technologies API-TOF reference mass standard solution. MS acquisition was conducted using drying gas flow rate of 9 L/min at 250°C, sheath gas flow rate of 11L/min at 325°C, and nebulizer pressure of 45 psig. The voltage gradient applied: capillary voltage, 3kV; nozzle voltage, 2kV; fragmentor, 100V; skimmer, 50V; radio frequency voltage applied to octopole (Oct 1 RF), 750V. Acquisition was conducted at an MS scan rate of 1.7 spectra/s and MS/MS scan of 3.4 spectra/s using narrow isolation width of ±1.3 m/z. Samples were injected at 8 µL over an 8.3 min gradient on the AdvanceBio Glycan Map 2.1 x 100 mm 2.7µm column at 35°C with a flow rate of 0.220 mL/min. The LC gradient only utilized LC/MS grade reagents when preparing mobile phases, A (88:12 H_2_O/acetonitrile (ACN) and B 90% ACN (aq). Both mobile phases were composed with 10 mM ammonium acetate and titrated to pH 6.85 using formic acid and ammonium hydroxide. The LC gradient was initially 100% B for 0.25 min and then ramped to 55% B at 2.5 min; 49% B at 4.5 min; 35% B at 5.5 min; 20% B at 6 min; held for 0.5 min; 15% B at 7 min; 100%B at 8.3 min followed by equilibration for 1.2 min.

### Human T Cell Preparation

Healthy donor T cells were acquired from the NIH blood bank (protocol 99-CC-0168). T cells were isolated by negative selection using the EasySep Human T cell isolation kit (Stemcell Technologies) and cryopreserved in 90% FBS and 10% DMSO until use. The cells were thawed in a 37°C water bath and cultured overnight in RPMI1640 medium containing 10% fetal bovine serum, 1% penicillin-streptomycin-glutamine,1% MEM non-essential amino acids solution, 15 mM HEPES, 1 mM sodium pyruvate and 55μM 2-mercaptoethanol as described previously (20). The T cells were then either used for staining by flowcytometry or for transwell migration assays as described above. TILs were isolated from patient tumor tissue obtained following surgical resection in the NOB at the NCI per the approved protocol, NCI-16C-0151, by the NCI-Institutional Review Board. Tumor tissue was digested using a cocktail of collagenase I and IV (Thermo Fisher Scientific, 200units/mL each) and Benzonase (Sigma, 0.5units/mL) in phenol red free IMDM medium (Thermo Fisher Scientific) following which they were mechanically sheared in a Medimachine (BD biosciences). The digest was collected into a tube with 1mL 2.5% BSA in PBS, topped with 0.25% BSA in PBS and then centrifuged to sediment the cells. Subsequently a Percoll gradient was then set up comprising of 30% Percoll (GE Healthcare Life Science), underlaid with 70% Percoll to separate the cells based on size. A clear band of TILs was obtained at the 70-30% Percoll interface that was then washed and cryopreserved in 90% FBS and 10% DMSO until further use. Upon thawing the cells were subject to similar conditions and procedures as the healthy donor T cells as described above.

### Statistical Analysis

All statistical analyses were performed using GraphPad Prism 7.0 software. The data are presented as mean ± standard error of the mean (SEM). Statistical significance was calculated in cases where two groups were being compared using a two-tailed Student’s *t*-test, comparisons with more than two groups were analyzed using analysis of variance (ANOVA). For *in vivo* murine tumor growth studies, tumor volumes/bioluminescence was converted to log scale (since tumors grow exponentially) and analyzed used one-way ANOVA, repeated measures with mixed effects model. *In vivo* murine survival studies were assessed using Kaplan-Meir analysis. *P*-values <= 0.05 were regarded significant. Concentration of GSK126 detected in each serum was normalized based on sample volume and each tissue sample was normalized to recorded sample weight following GSK126 quantification using calibration curve (R^2^ = 0.9998) generated with linear regression and no weighting.

## Results

### Primary Brain Tumors Are Enriched for CXCR3^+^ T Cells and Treatment of Human Glioma Cell Lines With GSK126 Reverses Histone Methylation Leading to Increased T Cell Chemoattraction

**Figure 1:**
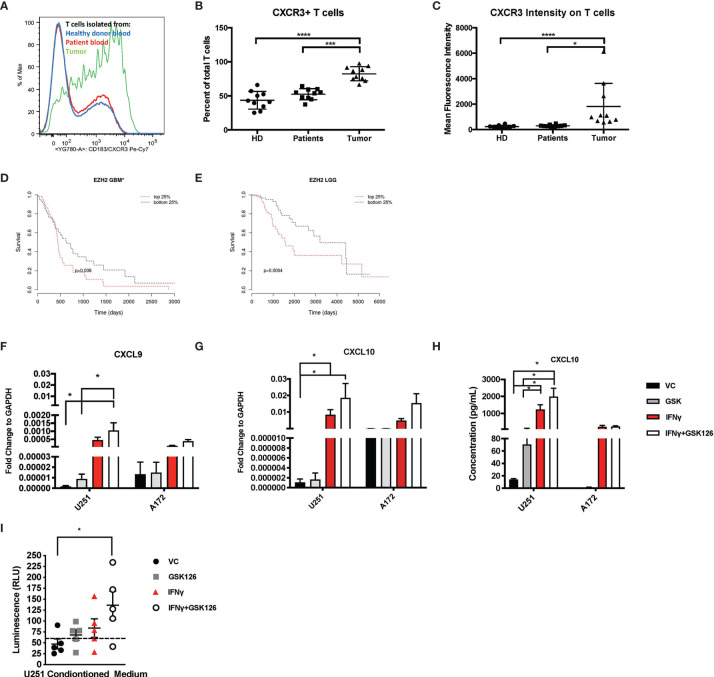
Brain Tumors are enriched for CXCR3^+^ T cells and treatment of glioma cell lines with GSK126 reverses histone methylation and increases expression of T cell chemoattractants. **(A)** Representative flow cytometry plot indicating the differences in the expression of CXCR3 on T cells isolated from healthy donor blood (blue), patient peripheral blood (red) and patient tumor tissue (green). **(B)** Graph representing the percentage of CXCR3^+^ T cells found in the blood of healthy donors (n=10), patient peripheral blood (n=10) versus those isolated from the tumor tissues of the same patients (n=10). Mean ± SEM is shown (p<=0.05 by one-way ANOVA). **(C)** The data represent the mean fluorescence intensity of CXCR3 expression as measured by flow cytometry on T cells obtained from donors and patients on **(B)** (mean ± SEM, p<=0.05 by one-way ANOVA). **(D, E)** Cistrome Cancer data showing that GBM patients **(D)** and low-grade glioma patients (LGG) **(E)** with higher expression (red) of EZH2 have a worse prognosis than those with a lower expression of EZH2. Cistrome Cancer is a comprehensive resource for predicted transcription factor (TF) targets and enhancer profiles in cancers. The prediction was from integrative analysis of TCGA expression profiles and public ChIP-seq profiles. **(F–G)** RT-PCR analysis examining the expression of CXCL9 **(F)** and CXCL10 **(G)** in human glioma cells lines U251 and A172 after treatment with either vehicle control (VC), IFNγ, GSK126 and GSK126+IFNγ for 24h (n=7 for U251 and n=3 for A172, mean ± SEM, p<=0.05 by two-way ANOVA). **(H)** Quantitation of CXCL10 protein secretion by ELISA using conditioned medium from human glioma cell lines U251 and A172 after treatment with either vehicle control (VC), GSK126 and/or IFNγ for 24h (n=3-4, mean ± SEM, p<=0.05 by two-way ANOVA). **(I)** T cell migration assay performed to assess movement of healthy donor T cells to tumor conditioned medium from human U251 cells, treated with VC, IFNγ, GSK126 and GSK126+IFNγusing CellTitre-Glo reagent (n=5, mean ± SEM, p<=0.05 by one-way ANOVA). The dotted line indicates luminescence reading for T cell movement to serum free medium control. *p < 0.05, ***p < 0.001 and ****p < 0.0001.

We examined and quantified the T cells from tumors of patients with CNS malignancies to establish that although immunologically “cold”, patients with CNS malignancies do have some basal T cell infiltration. Flow cytometry-based analysis of healthy donor T cells were compared to patient peripheral T cells and tumor infiltrating T cells and demonstrated that the T cells from the tumor had a higher expression of CXCR3 (**Figures 1A–C** and **Table 1**), highlighting the dependance on chemokine signaling, specifically CXCL9 and CXCL10 to traffic T cells from the periphery into the brain (**Figures 1A–C** and **Table 1**).

**Table 1:** Detailed description of patient and healthy donor characteristics for candidates in **Figure 1**.

	Healthy Donors (n = 10)	Patients (n = 10)
**Sex**		
Male	6	7
Female	4	3
**Age (years)**		
11-20	0	1
21-30	3	2
31-40	1	1
41-50	1	3
51-60	0	1
61-70	4	2
71-80	1	0
**Prior Treatment**		
Surgery		8
Radiation		6
Chemotherapy		4
Any 2 or more		6
Any 3 or more		3
**Tumor Type**		
Glioblastoma		3
Astrocytoma, grade-III		2
Anaplastic Oligodendroma		3
Astrocytoma, grade-II		1
Atypical Pilocytic		1

Epigenetic silencing of gene expression has been shown as one of the mechanisms by which solid tumor cells evade immune cells. EZH2 is a major contributor to epigenetic changes, most notably genomic (histone) methylation. We used Cistrome Cancer to analyze data from The Cancer Genome atlas (TCGA) and showed that both in GBM as well as low grade gliomas (LGGs), patients with higher EZH2 expression have a worse prognosis in comparison to patients with lower EZH2 expression (**Figures 1D, E**). These results suggest that EZH2 mediated gene silencing may play an important role in GBM tumor biology, influencing both cancer development as well as shaping the tumor-immune landscape.

We then tested if GSK126, a small molecule pharmacologic inhibitor of EZH2, can reverse histone methylation in brain tumor cells *in vitro*. We treated human GBM cell lines A172 and U251 with GSK126 in the presence and absence of IFNγ, a known inducer of CXCL9 and CXCL10 expression (**Supplementary Figures 1A, B**) (21). U251 cells displayed a greater decrease in H3K27me^3^ with GSK126 treatment compared to A172, indicating response in U251 but not the A172 cell line. Subsequent qPCR analysis revealed an additive effect of GSK126 and IFNγ treatment in increasing the expression of CXCL9 and CXCL10 in the U251 cell line and not the A172 cell line, corresponding to the changes seen in H3K27me^3^ upon treatment with GSK126 (**Figures 1F, G**). A similar pattern was also seen in CXCL10 protein expression as detected in conditioned medium from the U251 and A172 cell lines by ELISA (**Figure 1H**). Additionally, viability studies performed on these human GBM cell lines using CellTitre-Glo showed that treatment with GSK126 and/or the combination of GSK126 and IFNγ had no detrimental effect on cell growth *in vitro* (**Supplementary Figure 1C**).

In order to test the functional significance of the increased expression of CXCL9 and CXCL10 from the tumor cells treated with GSK126 and IFNγ, we performed a transwell migration assay using conditioned medium from U251 cells (since they responded to GSK126 treatment) and T cells isolated from five healthy donor PBMCs. We found that T cell migration to tumor conditioned medium from cells treated with the combination of GSK126 and IFNγ was significantly increased when compared to medium from vehicle treated cells (**Figure 1I**), the trend being additive of the single treatments.

Our data thus demonstrate that treatment of some human GBM cells with GSK126 *in vitro*, results in a reversal of H3K27me^3^ that allows increased expression of CXCL9 and CXCL10 when induced by IFNγ leading to increased migration of T cells to tumor conditioned medium.

### Treatment With GSK126 Decreases Intracranial Tumor Growth in Immunocompetent Mice

With the *in vitro* evidence in human U251 cells that treatment of tumor cells with GSK126 and IFNγ increased T cell chemoattraction, we evaluated the *in vivo* impact of treating immunocompetent tumor bearing mice with the drug, GSK126 in combination with an ICI, anti-PD-1 currently under investigation to treat patients with GBM by measuring the effects on tumor growth and survival. Based our *in vitro* data above and the fact that GSK126 is a non-cell type specific, global histone demethylating agent, we hypothesized that single agent treatment with the drug alone may not be sufficient to induce a significant anti-tumor effect *in vivo*. Combining it with an immune checkpoint inhibitor such as anti-PD-1 could be synergistic and enhance the anti-tumor effect of GSK126. To this end, we first treated murine GBM cell lines with GSK126 to examine changes in H3K27me^3^. Immunofluorescence analysis demonstrated that both GL261 and CT2A murine GBM cells responded to treatment with GSK126 and/or GSK126 and IFNγ, to decrease in H3K27me^3^ without having any detrimental effects on cell growth (**Supplementary Figures 2A–C**). Moving forward, we decided to use CT2A glioma cells for *in vivo* studies, since they better represent the human disease (22). The GL261 cell line is considered partially immunogenic with a high level of expression of MHC-I and has been shown to respond to immune checkpoint inhibition among many other modes of immunotherapy. Conversely, the CT2A cell line is considered to be less immunogenic, yet displaying important characteristics of human GBMs including tumor heterogeneity, invasiveness and stem like properties. For our *in vivo* studies, we first wanted to determine the if GSK126 had the ability to permeate the brain, to see if whether its action can be intratumoral or if it’s restricted to the periphery. Two cohorts of immunocompetent C57BL/6 (albino) mice implanted intracranially with CT2A tumors with treated systemically with either vehicle or GSK126. After 15 days of continuous treatment either with vehicle or GSK126, LC-MS analysis was performed on serum, tumor tissue and lumbar lymph nodes collected 2h, 6h and 10h following the final dose of drug treatment (**Supplementary Figures 2A, B**). GSK126 was detected both within the tumor as well as peripherally.

**Figure 2:**
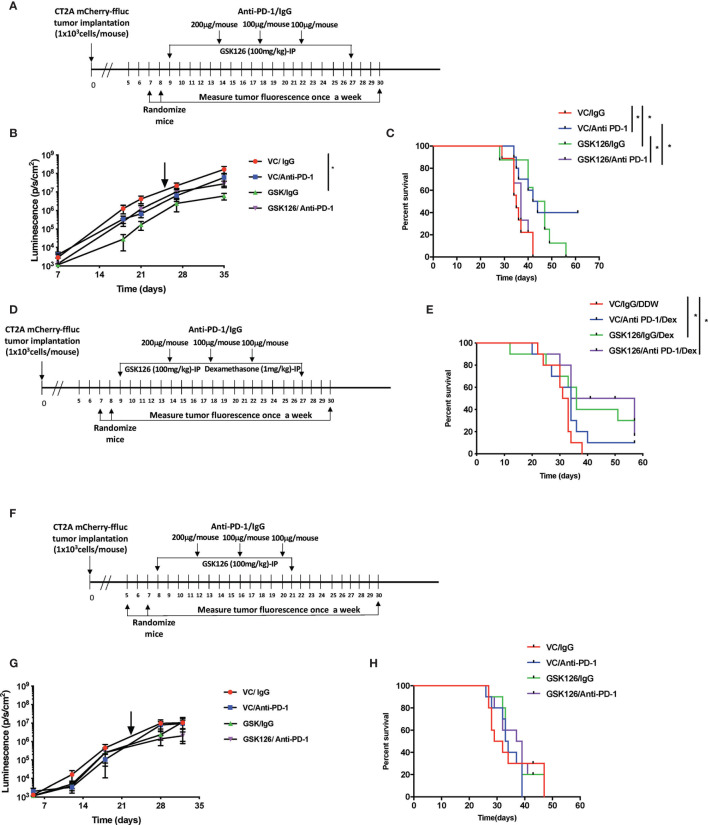
Treatment with GSK126 decreases intracranial tumor growth in immunocompetent mice. **(A)** Timeline indicating the intracranial tumor implantation and subsequent randomization and treatment regimens in immune competent C57BL/6 (albino) mice. **(B)** Graph showing tumor growth recorded as tumor luminescence over a 35-day period from date of tumor implantation in four cohorts of mice- VC/IgG treated (red), VC/Anti- PD-1 treated (blue), GSK126/IgG treated (green) and GSK126/Anti-PD-1 treated (purple) (n=10 per cohort, mean ± SEM, p<=0.05 by one-way ANOVA, repeated measures with mixed effects model on log transformed values). The arrow indicates the time point at which treatment was stopped. **(C)** Kaplan-Meir Curves for the experiment described in **(A)** (n=10 per cohort, p<=0.05 by log rank test). Median survival times are 35, 43, 44.5 and 37 days respectively for VC/IgG treated (red), VC/Anti- PD-1 treated (blue), GSK126/IgG treated (green) and GSK126/Anti-PD-1 treated (purple). **(D)** Timeline indicating the intracranial tumor implantation and subsequent randomization and treatment regimens in combination with dexamethasone in immune competent C57BL/6 (albino) mice. **(E)** Kaplan-Meir Curves for the experiment described in **(D)** (n=10 per cohort, p<=0.05 by log rank test). Median survival times are 32, 34, 36 and 45.5 days respectively for VC/IgG/DDW treated (red), VC/Anti- PD-1/Dex treated (blue), GSK126/IgG/Dex treated (green) and GSK126/Anti-PD-1/Dex treated (purple). **(F)** Timeline indicating the intracranial tumor implantation and subsequent randomization and treatment regimens in immune compromised SCID mice. **(G)** Graph indicating tumor growth recorded as tumor luminescence over a 35-day period from date of tumor implantation in four cohorts of mice- VC/IgG treated (red), VC/Anti- PD-1 treated (blue), GSK126/IgG treated (green) and GSK126/Anti-PD-1 treated (purple) (n=10 per cohort, mean ± SEM, p<=0.05 by one-way ANOVA, repeated measures with mixed effects model on log transformed values). **(H)** Kaplan-Meir Curves for the experiment described in **(G)**. (n=10 per cohort, p<=0.05 by log rank test). Median survival times are 30, 33.5, 33.5 and 38 days respectively for VC/IgG treated (red), VC/Anti- PD-1 treated (blue), GSK126/IgG treated (green) and GSK126/Anti-PD-1/Dex treated (purple). *p < 0.05.

Subsequently, four cohorts of C57BL/6 (albino) mice were injected intracranially with CT2A cells expressing firefly luciferase and mCherry reporters. As indicated in the timeline in **Figure 2A**, tumors were first detected 7 days post implantation by bioluminescence imaging. Subsequently, the mice were randomized and treated either with vehicle or GSK126 interperitoneally for 18 consecutive days alongside three doses of isotype control or anti-PD-1 antibody, respectively. Mice treated with GSK126 alone had a significantly slower tumor growth than mice treated with a combination of GSK126 and anti-PD-1 antibody (**Figure 2B**). In addition, we also observed that the cohort treated with GSK126 alone had a significant survival advantage over the combination group (**Figure 2C**). Interestingly 40% of the mice in the cohort treated with anti-PD-1 alone cleared their tumors giving them a survival benefit (**Figure 2C**). However, this finding should certainly be cautiously interpreted, given that results from clinical trials in GBM administered with ICI alone have not been very successful. The finding that treatment of tumor bearing mice treated with GSK126 along had significantly slower growth and better survival over those treated with the combination of GSK126 and anti-PD-1 led us to speculate on the possible reasons. One plausible explanation based on preliminary histopathologic analysis of a small sample of tumors from these mice indicated that combinatorial treatment may have caused inflammation due to increased cytotoxic T cell accumulation, that results in cerebral edema and eventual death. However, it is important to recognize that definitive assessment of edema in mice, especially from tissue slides is challenging due to the lack of specific markers. A similar phenomenon has also been evidenced in patients with CNS malignancies where an inflammatory response cannot be distinguished from tumor progression (23).

To address this possibility, we treated intracranial tumor bearing mice with a corticosteroid, dexamethasone, commonly used to treat peritumoral edema in patients with GBM. Dexamethasone was given in combination with GSK126 and anti-PD-1 antibody. As depicted in the timeline (**Figure 2D**), dexamethasone treatment was added to the regime alongside treatment with GSK126 and anti-PD-1 after confirming the establishment of tumors by bioluminescence imaging and subsequent randomization. This ensures that the immunosuppressive effects of dexamethasone are minimized as shown previously by our group and others (20, 24). The results demonstrated that treatment with dexamethasone rescued the mice treated with the combination of GSK126 and anti-PD-1 antibody giving them a significant survival benefit over the control group (**Figure 2E**). An additional survival benefit was also seen in the cohort treated with GSK126 and dexamethasone (**Figure 2E**). On the other hand, we also observe a loss in survival benefit from anti-PD-1 treatment alone with the addition of dexamethasone treatment regimen. While the reason for this decrease in survival remains to be investigated in detail, previous studies in our group have shown that treatment with anti-PD-1 is unable to rescue defects in T cell proliferation induced by dexamethasone (20). Hence it is possible that this makes mice treated with anti-PD-1 and dexamethasone more susceptible to decreased survival.

An important component of the GBM tumor microenvironment are myeloid cells. Since GSK126 is a global histone demethylating agent, we wanted to verify that the *in vivo* differences in tumor growth and survival due to GSK126 treatment were mediated largely by lymphocytes, rather than myeloid cells. Hence, we implanted CT2A firefly luciferase-mCherry cells intracranially into four cohorts of severe combined immunodeficient (SCID) mice that lack B and T cells but have a functional innate immune system (25). After randomization, the mice were treated either with vehicle or GSK126 interperitoneally alongside three doses of isotype control or anti-PD-1 antibody respectively (**Figure 2F**). No significant differences were seen in the rate of tumor growth and survival between the different cohorts, indicating that any survival advantage or diminished tumor growth observed in previous experiments involving immunocompetent mice was mainly attributable to lymphocytes (**Figures 2G, H**).

### Combinatorial Treatment of GSK126 and Anti PD-1 Antibody Decreases Tumor Growth and Improves Survival in a Sub-Cutaneous Model of Murine GBM

**Figure 3:**
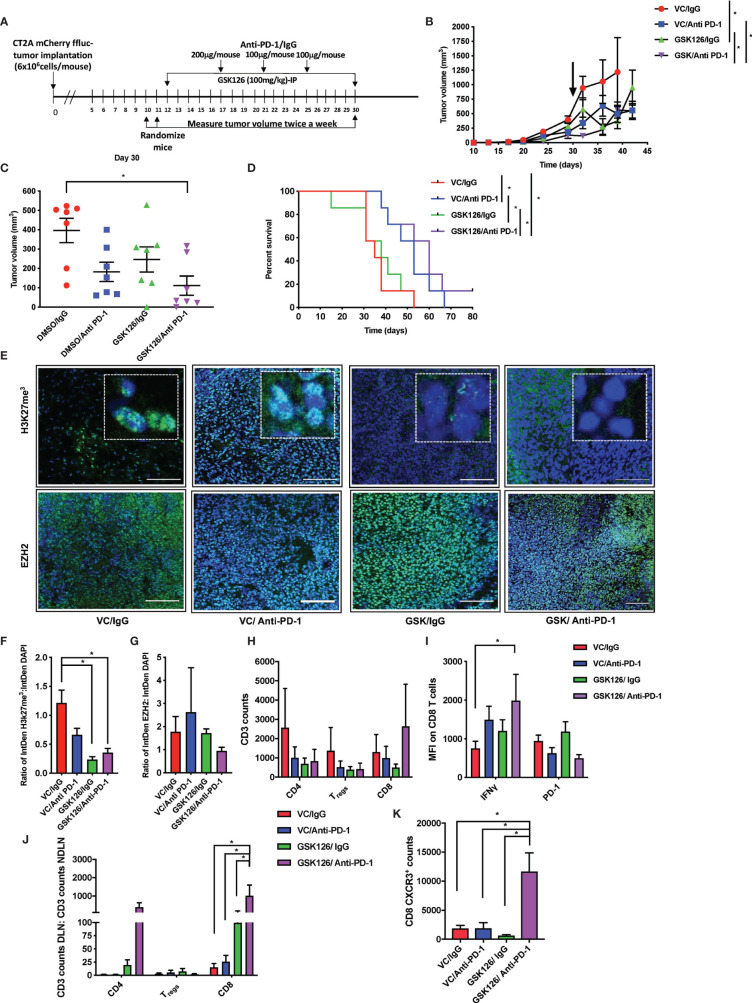
Combinatorial treatment of GSK126 and anti PD-1antibody decreases tumor growth and improves survival in sub-cutaneous tumors. **(A)** Timeline indicating the sub-cutaneous tumor implantation and subsequent randomization and treatment regimens in immune competent C57BL/6 (albino) mice. **(B)** Graph showing tumor growth recorded as tumor volume over a 45-day period from date of tumor implantation in four cohorts of mice- VC/IgG treated (red), VC/Anti- PD-1 treated (blue), GSK126/IgG treated (green) and GSK126/Anti-PD-1 treated (purple) (n=7 per cohort, mean ± SEM, p<=0.05 by one-way ANOVA, repeated measures with mixed effects model on log transformed values). The arrow indicates the time point at which treatment was stopped. **(C)** Comparison of tumor volume 30 days after tumor implantation following termination of treatment either with GSK126, anti-PD-1 antibody or both alongside the control cohort from **(B)** (n=7 per cohort, mean ± SEM, p<=0.05 by one-way ANOVA). **(D)** Kaplan-Meir Curves for the experiment described in **(A)** (n=7 per cohort, p<=0.05 by log rank test). Median survival times are 35, 50, 38 and 60 days respectively for VC/IgG treated (red), VC/Anti- PD-1 treated (blue), GSK126/IgG treated (green) and GSK126/Anti-PD-1 treated (purple). **(E)** Representative immunofluorescence images for staining with H3K27me^3^ and EZH2 performed on two consequent tumor sections respectively from tumor tissue obtained from a separate experiment similar to that described in(B) but terminated 27 days after tumor implantation. Positive staining for H3K27me^3^ and EZH2 (green) overlaps with DAPI nuclear counterstain (blue) (20X magnification). Scale bar denotes 100µm. **(F–G)** Quantitative analysis of H3K27me^3^ and EZH2 as obtained from **(E)** expressed as a ratio to DAPI positive nuclei (n=3 per cohort, mean ± SEM, p<=0.05 from one-way ANOVA). **(H)** Flow cytometry analysis of CD3^+^ T cells isolated from subcutaneous tumors of mice described in **(E)** indicates that GSK126 and anti-PD-1combination treated mice had a greater number of CD8^+^ T cells. These cells were gated from mCherry^-^ and CD3^+^ T cells (n=6, mean ± SEM, two- way ANOVA). **(I)** Flow cytometry analysis of the median fluorescence intensity (MFI) of IFNγ and PD-1 expression on CD8^+^ T cells from tumor lymphocytes obtained from **(E)** and gated from **(H)** demonstrates that combination treatment group had a significant expression of IFNγ (n=6, mean ± SEM, p<=0.05 by two-way ANOVA). **(J)** Flow cytometry analysis of CD3^+^ T cells isolated from the DLN and NDLN expressed as a ratio indicates that GSK126 and anti-PD-1 treated mice had a significant increase in CD8^+^ T cells (n=6, mean ± SEM, p<=0.05 by two-way ANOVA). **(K)** Flow cytometry analysis of CXCR3 expression of CD8 T cells isolated from the DLN indicates that GSK126 and anti-PD-1 treated mice had a greater CXCR3^+^ T cells (n=6, mean ± SEM, p<=0.05 by one-way ANOVA). *p < 0.05.

Following the finding in the intracranial model that GSK126 has an anti-tumor function that is enhanced by combination treatment with anti-PD-1 and dexamethasone, we wanted to investigate the mode of action of GSK126 both as a single agent as well as in combination with anti-PD-1, *in vivo*. For these studies we decided to use a subcutaneous tumor model of glioblastoma using the CT2A cell lines. Although the subcutaneous tumor model has its limitations in the study of GBM, mainly the absence of physical barriers such as the cranial vault, the BBB and the BCSFB, in the context of this study, since GSK126 is found to permeate the BBB use of the sub-cutaneous model is advantageous since it would eliminate study limiting concerns of excessive immune activity in the tumor as a result of treatment. Four cohorts of C57Bl/6 (albino) mice were injected subcutaneously with syngeneic CT2A murine GBM cells that stably express mCherry and firefly luciferase. As shown in the timeline in **Figure 3A**, the tumors were palpable starting 7 days after tumor injection. Subsequently, the mice were randomized and treated either with vehicle or GSK126 interperitoneally for 18 consecutive days alongside three doses of isotype control or anti-PD-1 antibody, respectively. Growth of tumors treated with the combination of GSK126, and anti-PD-1 antibody was found to be significantly slower than the control, vehicle treated tumors as well as tumors from mice treated with each of the drugs as single agents (**Figures 3B, C**). This decrease in tumor growth also provided a significant survival benefit for these mice (**Figure 3D**). It is important to remember that the absence of the BBB and BCSFB allows increased access of anti-PD-1 antibodies to the tumor in the sub cutaneous tumor model. We then repeated this regimen in four separate cohorts of C57BL/6 (albino) mice and terminated the experiment 27 days post tumor injection after having completed 15 days of consecutive treatment with vehicle/GSK126 and all three doses of isotype/anti-PD1 respectively in order to investigate if the differences in tumor growth could be attributed to changes in immune microenvironment within the tumor arising as result of decrease in intratumoral H3K27me^3^. Immunofluorescence testing of a sample of three tumors from each of the cohorts confirmed that the tumors from the cohorts of mice treated with GSK126 had a significant decrease in H3K27me^3^ as compared to the vehicle treated tumors but no significant decrease in expression of EZH2 between the groups (**Figures 3E–G**). Thus, we believe that the effect of the drug on the tumor is specific to its documented ability to inhibit EZH2 mediated histone methylation. Flow cytometry-based analysis of tumor tissue from the six remaining mice in each cohort revealed an increase in the number of CD8^+^ T cell infiltrates in mice treated with GSK126 and anti-PD-1 which although not statistically significant points to a trend towards increase in cytotoxic T cell infiltration of the tumor (**Figure 3H**). Importantly, a significant increase in IFNγ expression was observed on these tumor-infiltrating CD8^+^ T cells as compared to the vehicle treated tumors (**Figure 3I** and **Supplementary Figure 3D**). Interestingly, we also found a significant increase in CD8^+^ T cells in the tumor draining lymph nodes (DLN) (the inguinal lymph node from the flank where the tumor was implanted) as compared to the non-draining lymph nodes (NDLN) (the inguinal lymph node from the flank opposite to the one where the tumor was implanted) in the mice treated with the combination regimen (**Figure 3J**). This, alongside a significant increase in CXCR3 expression on the CD8^+^ T cells within the draining lymph node, suggest that the T cells were primed for migration, potentially to the tumor (**Figure 3K**). This finding is especially germane to the lymph nodes since they are the centers for T cell activation and expansion, receiving cues that direct them to the tumor. Another key component of the micro-environment of the lymph node are the myeloid cells. While treatment with GSK126 had no significant impact on the recruitment of macrophages, monocytes and/or dendritic cells *in vivo* either in the presence or absence of anti-PD-1 treatment (**Supplementary Figures 3E, F**), in the tumor draining lymph node, their contribution to the stimulation and further mobilization of T cells is likely.

In order to verify that the activity of immune cells seen in the tumor and draining lymph node were specific to the ability of the drug, GSK126 to enter these tissues, we performed LC-MS analysis on serum and tumor tissue collected at three time points 2h, 6h and 10h following the final dose of drug treatment from two cohorts of C57BL/6 mice bearing subcutaneous CT2A tumors and treated for 15 days with either vehicle or GSK126. The drug was detected clearly above background in serum as well as tumor tissue and in the lymph nodes (**Supplementary Figures 3G, H**).

### Treatment With GSK126 Selectively Enhances Expression of T Cell Chemotactic Factors From Murine Glioma Cells Without Directly Affecting T Cell Maturation and Proliferation

**Figure 4:**
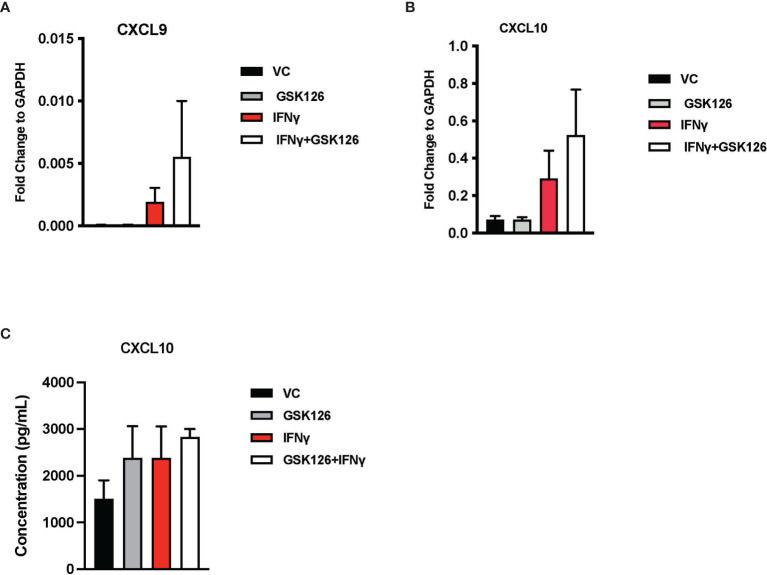
Treatment with GSK126 selectively enhances expression of T cell chemotactic factors from murine glioma cells without directly affecting T cell maturation and proliferation. **(A, B)** RT-PCR analysis examining the expression of CXCL9 **(A)** and CXCL10 **(B)** in murine glioma cells CT2A following a 48h pre-treatment with GSK126 and a subsequent treatment with IFNγ for another 24h (n=3-4, mean ± SEM). **(C)** Quantitation of CXCL10 protein secretion by ELISA using conditioned medium from CT2A cells pre-treatment with GSK126 and a subsequent treatment with IFNγ as described in **(A)** (n=3, mean ± SEM).

Results from our *in vivo* studies thus far have shown that GSK126 in combination with anti-PD-1, promotes the infiltration of activated T cells within the tumor perhaps trafficking from the draining lymph nodes where there is evidence of accumulation of CXCR3^+^ T cells. In order to better understand the mode of action of GSK126, beyond its ability to inhibit EZH2 mediated H3K27me^3^, we pre-treated murine CT2A mCherry firefly luciferase expressing cells with GSK126 for 48h, prior to treatment with IFNγ in an attempt to recreate the effect of treating tumors *in vivo* with the combination of GSK126 and anti-PD-1 antibody. qPCR analysis revealed an increase in the expression of CXCL9 and CXCL10 chemokines from these tumor cells (**Figures 4A, B**). A trend towards increased expression of CXCL10 protein was also detected in conditioned media from cells treated with the combination of GSK126 and IFNγ (**Figure 4C**). We then wanted to verify if GSK126 also had a direct effect on T cells. To this end, we stimulated primary murine T cells with anti-CD3/anti-CD28 antibodies then treated with GSK126 in the presence or absence of IFNγ. We found that GSK126 had no effect on T cell maturation or proliferation (**Supplementary Figures 4A-C**). We also examined the expression of CXCL9, CXCL10 and CXCR3 on these T cells and found no significant difference in their expression (**Supplementary Figure 4D**). It is therefore likely that the increased expression of CXCR3 seen on the T cells in draining lymph node *in vivo* is in response to paracrine CXCL9 and CXCL10 signals possibly from the tumor.

Taken together, our data suggest that one of the primary targets of GSK126 are the tumor cells, enhancing their expression of T cell chemoattractants without directly affecting the T cell activity.

## Discussion

Glioblastomas are commonly referred to as “cold” tumors due to a paucity of infiltrating immune cells. The presence of a hostile tumor micro-environment, lack of immune cell trafficking to the brain and presence of physical barriers such as the BBB and/or the BCSF barrier are the primary contributors making GBMs “immune deserts” (26). Therapeutic strategies that will break this pattern of immune suppression and allow immune cells, specifically cytotoxic T lymphocytes to carry out their tumor killing function may help augment immunotherapy strategies for this patient population. Epigenetic gene silencing is one of the mechanisms by which tumor cells “hijack” the immune system. In this study, we have shown that a small molecule inhibitor of histone methylation GSK126, can reverse this immune evasion, leading to a decrease in tumor growth and improvement in survival in pre-clinical murine models of GBM. In addition, the combination of GSK126 with an anti-PD-1 antibody enhances the efficacy of treatment.

GSK126 is a histone demethylation agent that can either derepress the expression of genes that promote anti-tumor immune responses or conversely increase the expression of genes that are inhibitory to anti-tumor immune responses, since its activity is not specific for a given cell type. It is thus likely that its effects are not limited to tumor cells. Other cells within the stroma may also undergo changes in response to treatment with this drug. This provides a potential explanation for the finding that GSK126 alone is limited in producing durable responses in mouse models of GBM. Interestingly, in mice, we also observed a tissue-based difference in response to treatment with anti-PD-1 and the combination of anti-PD-1 and GSK126. In the subcutaneous GBM tumor model, the contribution of anti-PD-1 treatment in the combination group may be more than GSK126 on survival of the mice although, not on immune cell trafficking to the tumor. In comparison, in the intracranial model, our results suggest particularly in combination with dexamethasone, that the contribution of GSK126 to the efficacy of the combination is greater. We suspect that the subcutaneous setting without the BBB and the BCSFB, anti-PD-1 antibodies are readily delivered to the tumor, maximizing its ability to act on tumor infiltrating immune cells as well as resident cells to mount a stronger anti-tumor response. In contrast, in the intracranial setting, the action of anti-PD-1 antibodies is largely limited to impacting immune cells in the periphery. However, GSK126 permeates into the intra-cranial tumor as our data suggest thereby allowing the drug to act in the periphery and within the tumor. Therefore, for intracranial tumors like GBM, combining drugs like GSK126 with an ICI like an anti-PD-1 may enhance immune response by augmenting chemokine release in both the intracranial tumor and the draining lymph nodes thereby causing both an increase in activated immune cells and improved trafficking to the tumor. Despite the inability of the anti-PD-1 antibodies to adequately cross the BBB (27), the systemic activity of these agents was sufficient to generate anti-tumor immune response that resulted in prolonged progression free survival in the intracranial model. Although encouraging, these data must be considered in the context of the results in clinical trials in GBM patients where administration of ICI alone has not been very successful.

Given the need to introduce corticosteroids into the animal model to see a survival benefit, translation of the combinatorial regimen of GSK126 and anti-PD-1 for clinical testing should proceed with caution. Prolonged immune activation resulting from treatment with GSK126 and anti-PD-1 may lead to uncontrolled inflammation and edema. While corticosteroids are often used to resolve the situation, careful monitoring of patients given this regimen is necessary (28). Additionally, the timing of dexamethasone may be critical to the success of immunotherapy in patients with primary brain tumors. Previous studies by our group and others have shown in murine models of GBM, that appropriately timing the administration of dexamethasone can prevent blunting of immune responses (20, 24). With the addition of dexamethasone to the treatment regimen to prevent excessive brain edema, we observed a significant improvement in survival of mice treated with GSK126 and anti-PD-1 antibody.

Our study thus demonstrates co-operative efficacy of GSK126 with ICI. These findings are in concert with other studies conducted in systemic solid cancers such as prostate cancer, melanoma and ovarian cancer that demonstrated a significant therapeutic benefit of from the combination of GSK126 and ICIs in decreasing tumor growth and increasing T cell trafficking to the tumor. Therefore, with the caveat that monitoring of intracranial immune response is critical, our results support testing systemic administration of GSK126 with ICIs in patients with primary intracranial cancers.

## Data Availability Statement

The datasets presented in this study can be found in online repositories. The names of the repository/repositories and accession number(s) can be found below: Metabolomics Workbench [Project ID PR001160; DOI: 10.21228/M8RT34].

## Ethics Statement

The studies involving human participants were reviewed and approved by National Cancer Institute-Institutional Review Board. The patients/participants provided their written informed consent to participate in this study. The animal study was reviewed and approved by National Cancer Institute-Bethesda Animal Care and Use Committee.

## Author Contributions

NR designed research studies, conducted experiments, acquired and analyzed data, and wrote the manuscript. HS also conducted the experiments, acquired and analyzed the data. SF, HC, M-KH, TD, CR, JJ, WZ, HS, MZ, and DD contributed to conducting specific experiments and acquiring data thereby obtained. ML contributed data and edited the manuscript. AG designed some experiments, contributed data and edited the manuscript. MG led the studies, analyzed data, and helped write the manuscript. All authors contributed to the article and approved the submitted version.

## Funding

This research was supported in part by the Intramural Research Program of the NIH, NCI.

## Conflict of Interest

The authors declare that the research was conducted in the absence of any commercial or financial relationships that could be construed as a potential conflict of interest.

## Supplementary Material

Supplementary Figure 1 GSK126 reverses histone methylation in human glioma cell lines without affecting cell growth in vitro. **(A)** Representative images of immunofluorescence staining of human glioma cell lines A172, and U251 stained for H3K27me3 in presence and absence of IFNγ and/or GSK126. H3K27me3 is a nuclear stain (green) that overlaps with DAPI nuclear counterstain (blue) (20X magnification) Scale bar denotes 200μM. **(B)** Quantitative analysis of fluorescence intensity of H3K27me3 staining relative to the number of nuclei (n=2-3, mean ± SEM, one-way ANOVA). **(C)** Cell Titreglo analysis of cell growth/survival of human glioma lines A172 and U251 over 3 days in culture in serum-free medium after treatment with either with vehicle control (VC), IFNγ, GSK126 and GSK126+IFNγ (n=3, mean ± SEM, two-way ANOVA).

Supplementary Figure 2 GSK126 crosses the blood-brain barrier to reach intracranial tumors and can act both peripherally and intratumorally. **(A, B)** LC-MS based quantitation from serum **(A)**, tumor tissue and lumbar lymph nodes **(B)** isolated from mice implanted intracranially with murine gliomas and treated with GSK126 shows that the drug can be detected above background within the tumor and peripherally (n=3 for per cohort 2h time point and n=2 per cohort for 6 and 10h time point, mean ± SEM, p<=0.05 by oneway ANOVA for **A** and two-way ANOVA for **B**).

Supplementary Figure 3 H3k27me3 methylation is reversed with the addition of GSK126 in murine glioma cells *in vitro* and GSK126 can penetrate the tumor as well as lymph nodes *in vivo*. **(A)** Representative images of immunofluorescence staining of murine glioma cell lines GL261 and CT2A mCherry-ffluc stained for H3K27me3 in presence and absence of IFNγ and/or GSK126. H3K27me3 is a nuclear stain (green) that overlaps with DAPI nuclear counterstain (blue) (10X magnification). Scale bar denotes 200μm. **(B)** Quantitative analysis of fluorescence intensity of H3K27me3 staining relative to the number of nuclei (n=3, mean ± SEM, one-way ANOVA). **(C)** Cell Titre-glo analysis of cell growth/survival of murine glioma lines GL261 and CT2A mCherry ffluc over 3 days in culture in serum-free medium after treatment with either vehicle control (VC), GSK126 and/or IFNγ for 24h (n=3, mean ± SEM, two-way ANOVA). **(D)** Flow cytometry analysis of the median fluorescence intensity (MFI) of IFNγ and PD-1 expression on CD4+ T cells from tumor lymphocytes obtained from Figure 2E and gated from Figure 2H demonstrates that combination treatment group had no significant effect on expression of IFNγ and anti-PD-1(n=6, mean ± SEM, p<=0.05 by two-way ANOVA). **(E, F)** Flow cytometry analysis of myeloid cells in the draining lymph nodes obtained from mice with subcutaneous tumors in described in Figure 2E, gated from CD3- CD11b+ cells in E and CD3- CD11c+ cells in F shows no significant differences with GSK and/or anti-PD-1 treatment [n=6, mean ± SEM, two-way ANOVA **(E)** and one-way ANOVA **(F)**]. **(G, H)** LC-MS based quantitation from serum **(G)**, tumor tissue and lymph nodes **(H)** isolated from mice implanted subcutaneously with murine gliomas and treated with GSK126 shows that the drug can be detected above background within the tumor and peripherally (n=3 for per cohort 2h time point and n=2 per cohort for 6 and 10h time point, mean ± SEM, p<=0.05 by one-way ANOVA for **G** and two-way ANOVA for **H**).

Supplementary Figure 4 GSK126 has no direct effect on T cell maturation and proliferation. **(A, B)** Flow cytometry- based analysis of T cell maturation subsets for CD4T cells **(A)** and CD8T cells **(B)** stimulated using anti-CD3/ anti-CD28 antibodies and treated with GSK126, IFNγ or the combination of the two (n=5 mice, mean ± SEM, one-way ANOVA). **(C)** Flow cytometry- based analysis of T cell proliferation following stimulation using anti-CD3/ anti-CD28 antibodies and treatment with GSK126, IFNγ or the combination of the two (n=2 mice, mean ± SEM, one-way ANOVA). (D) qPCR analysis of the expression of CXCL9, CXCL10 and CXCR3 in T cells following stimulation using anti-CD3/ anti-CD28 antibodies and treatment with GSK126, IFNγ or the combination of the two (n=5 mice, mean ± SEM, one-way ANOVA).

## References

[1] Ostrom QT Cioffi G Gittleman H Patil N Waite K Kruchko C. CBTRUS Statistical Report: Primary Brain and Other Central Nervous System Tumors Diagnosed in the United States in 2012-2016. Neuro Oncol (2019) 21:V1–V100. 10.1093/neuonc/noz150 31675094 PMC6823730

[2] Agnihotri S Burrell KE Wolf A Jalali S Hawkins C Rutka JT. Glioblastoma, A Brief Review of History, Molecular Genetics, Animal Models and Novel Therapeutic Strategies. Arch Immunol Ther Exp (Warsz) (2013) 61:25–41. 10.1007/s00005-012-0203-0 23224339

[3] Le Rhun E Preusser M Roth P Reardon DA van den Bent M Wen P. Molecular Targeted Therapy of Glioblastoma. Cancer Treat Rev (2019) 80:101896. 10.1016/j.ctrv.2019.101896 31541850

[4] Carson MJ Doose JM Melchior B Schmid CD Ploix CC. CNS Immune Privilege: Hiding in Plain Sight. Immunol Rev (2006) 213:48–65. 10.1111/j.1600-065X.2006.00441.x 16972896 PMC2633103

[5] Mrdjen D Pavlovic A Hartmann FJ Schreiner B Utz SG Leung BP. High-Dimensional Single-Cell Mapping of Central Nervous System Immune Cells Reveals Distinct Myeloid Subsets in Health, Aging, and Disease. Immunity (2018) 48:380–95.e6. 10.1016/j.immuni.2018.01.011 29426702

[6] Smolders J Heutinck KM Fransen NL Remmerswaal EBM Hombrink P ten Berge IJM. Tissue-Resident Memory T Cells Populate the Human Brain. Nat Commun (2018) 9:4593. 10.1038/s41467-018-07053-9 30389931 PMC6214977

[7] Pasciuto E Burton OT Roca CP Lagou V Rajan WD Theys T. Microglia Require Cd4 T Cells to Complete the Fetal-To-Adult Transition. Cell (2020) 182:625–40.e24. 10.1016/j.cell.2020.06.026 32702313 PMC7427333

[8] Louveau A Harris TH Kipnis J. Revisiting the Mechanisms of CNS Immune Privilege. Trends Immunol (2015) 36:569–77. 10.1016/j.it.2015.08.006 PMC4593064 26431936

[9] Sampson JH Maus MV June CH. Immunotherapy for Brain Tumors. J Clin Oncol (2017) 35:2450–6. 10.1200/JCO.2017.72.8089 28640704

[10] Ridley A Cavanagh JB. Lymphocytic Infiltration in Gliomas: Evidence of Possible Host Resistance. Brain (1971) 94:117–24. 10.1093/brain/94.1.117 5552158

[11] Mohme M Schliffke S Maire CL Runger A Glau L Mende KC. Immunophenotyping of Newly Diagnosed and Recurrent Glioblastoma Defines Distinct Immune Exhaustion Profiles in Peripheral and Tumor-Infiltrating Lymphocytes. Clin Cancer Res (2018) 24:4187–200. 10.1158/1078-0432.CCR-17-2617 29444930

[12] Jackson C Ruzevick J Phallen J Belcaid Z Lim M. Challenges in Immunotherapy Presented by the Glioblastoma Multiforme Microenvironment. Clin Dev Immunol (2011) 2011:732413. 10.1155/2011/732413 22190972 PMC3235820

[13] Wei J Chen P Gupta P Ott M Zamler D Kassab C. Immune Biology of Glioma-Associated Macrophages and Microglia: Functional and Therapeutic Implications. Neuro Oncol (2020) 22:180–94. 10.1093/neuonc/noz212 PMC7442334 31679017

[14] Gabrusiewicz K Rodriguez B Wei J Hashimoto Y Healy LM Maiti SN. Glioblastoma-Infiltrated Innate Immune Cells Resemble M0 Macrophage Phenotype. JCI Insight (2016) 1:0–19. 10.1172/jci.insight.85841 PMC4784261 26973881

[15] Dunn GP Dunn IF Curry WT. Focus on TILs: Prognostic Significance of Tumor Infiltrating Lymphocytes in Human Glioma. Cancer Immun (2007) 7:1–16. 17691714 PMC2935751

[16] Dunn GP Bruce AT Ikeda H Old LJ Schreiber RD. Cancer Immunoediting: From Immuno- Surveillance to Tumor Escape. Nat Immunol (2002) 3:991–8. 10.1038/ni1102-991 12407406

[17] Zingg D Arenas-Ramirez N Sahin D Rosalia RA Antunes AT Haeusel J. The Histone Methyltransferase Ezh2 Controls Mechanisms of Adaptive Resistance to Tumor Immunotherapy. Cell Rep (2017) 20:854–67. 10.1016/j.celrep.2017.07.007 28746871

[18] Huang S Wang Z Zhou J Huang J Zhou L Luo J. EZH2 Inhibitor GSK126 Suppresses Antitumor Immunity by Driving Production of Myeloid-Derived Suppressor Cells. Cancer Res (2019) 79:2009–20. 10.1158/0008-5472.CAN-18-2395 30737232

[19] Peng D Kryczek I Nagarsheth N Zhao L Wei S Wang W. Epigenetic Silencing of TH1-Type Chemokines Shapes Tumour Immunity and Immunotherapy. Nature (2015) 527:249–53. 10.1038/nature15520 PMC4779053 26503055

[20] Giles AJ Hutchinson MKND Sonnemann HM Jung J Fecci PE Ratnam NM. Dexamethasone-Induced Immunosuppression: Mechanisms and Implications for Immunotherapy. J Immunother Cancer (2018) 6:1–13. 10.1186/s40425-018-0371-5 29891009 PMC5996496

[21] Metzemaekers M Vanheule V Janssens R Struyf S Proost P. Overview of the Mechanisms That may Contribute to the non-Redundant Activities of Interferon-Inducible CXC Chemokine Receptor 3 Ligands. Front Immunol (2018) 8:1970. 10.3389/fimmu.2017.01970 29379506 PMC5775283

[22] Oh T Fakurnejad S Sayegh ET Clark AJ Ivan ME Sun MZ. Immunocompetent Murine Models for the Study of Glioblastoma Immunotherapy. J Transl Med (2014) 12:1–10. 10.1186/1479-5876-12-107 24779345 PMC4012243

[23] Ranjan S Quezado M Garren N Boris L Siegel C Lopes Abath Neto O. Clinical Decision Making in the Era of Immunotherapy for High Grade-Glioma: Report of Four Cases. BMC Cancer (2018) 18:239. 10.1186/s12885-018-4131-1 29490632 PMC5831705

[24] Iorgulescu JB Gokhale PC Speranza MC Eschle BK Poitras MJ Wilkens MK. Concurrent Dexamethasone Limits the Clinical Benefit of Immune Checkpoint Blockade in Glioblastoma. Clin Cancer Res (2020) 27:1–13. 10.1158/1078-0432.ccr-20-2291 PMC8034990 33239433

[25] Vladutiu AO. The Severe Combined Immunodeficient (SCID) Mouse as a Model for the Study of Autoimmune Diseases A. Clin Exp Immunol (1993) 93:1–8. 10.1111/j.1365-2249.1993.tb06488.x 8324894 PMC1554753

[26] Weiss T Puca E Silginer M Hemmerle T Pazahr S Bink A. Immunocytokines Are a Promising Immunotherapeutic Approach Against Glioblastoma. Sci Transl Med (2020) 12(564):eabb2311. 10.1126/scitranslmed.abb2311 33028706

[27] Kim M Kizilbash SH Laramy JK Gampa G Parrish KE Sarkaria JN. Barriers to Effective Drug Treatment for Brain Metastases: A Multifactorial Problem in the Delivery of Precision Medicine. Pharm Res (2018) 35:177. 10.1007/s11095-018-2455-9 30003344 PMC6700736

[28] Dietrich J Rao K Pastorino S Kesari S. Corticosteroids in Brain Cancer Patients: Benefits and Pitfalls. Expert Rev Clin Pharm (2011) 4:233–42. 10.1586/ecp.11.1 PMC3109638 21666852

