# Supplementary figures and images for: Reversing Epigenetic Gene Silencing to Overcome Immune Evasion in CNS Malignancies
Source: Front Oncol. 2021 Jul 15;11:719091. doi: 10.3389/fonc.2021.719091 (PMC8320893; doi:10.3389/fonc.2021.719091)

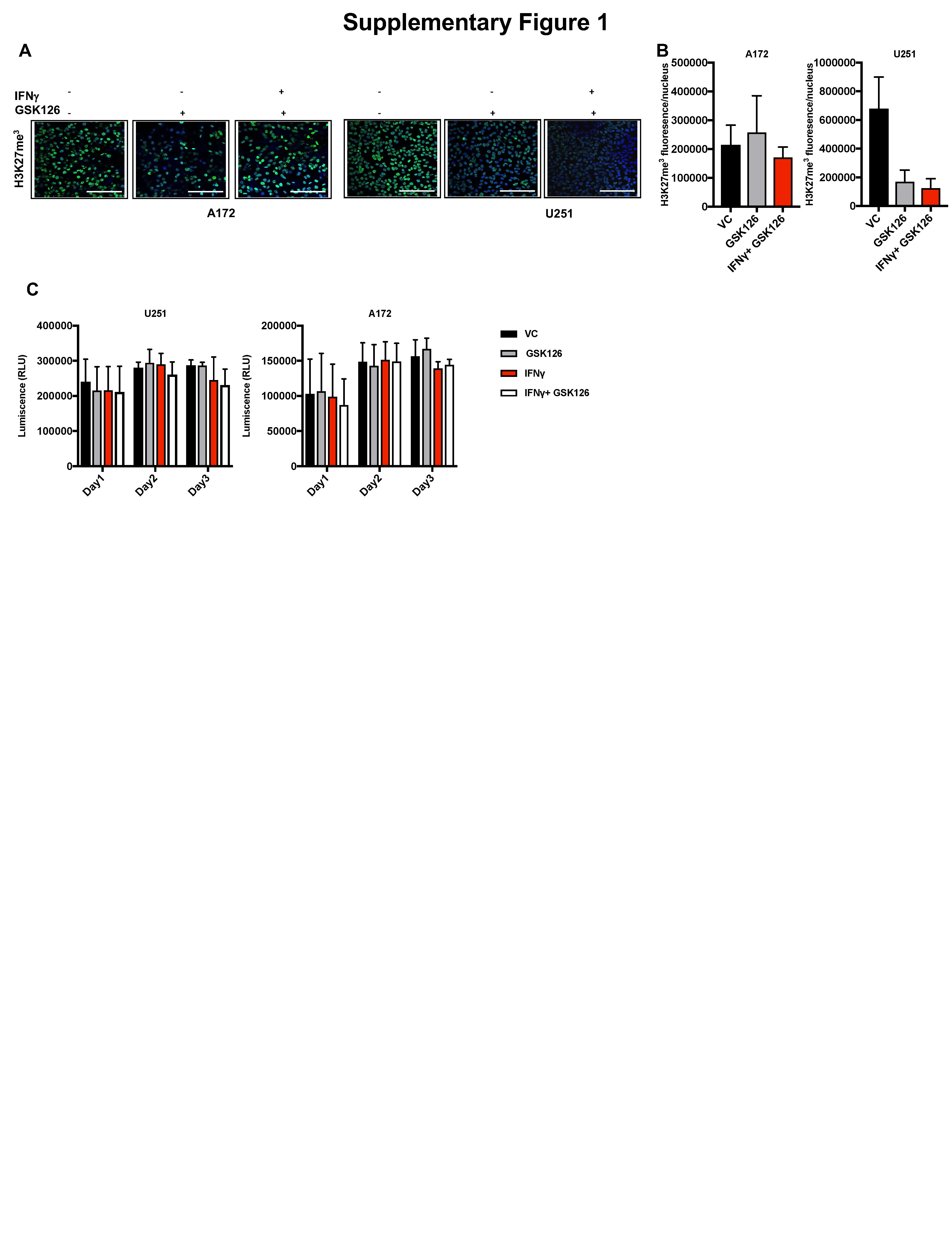

Supplement: Supplementary Figure 1 — GSK126 reverses histone methylation in human glioma cell lines without affecting cell growth in vitro. (A) Representative images of immunofluorescence staining of human glioma cell lines A172, and U251 stained for H3K27me3 in presence and absence of IFNγ and/or GSK126. H3K27me3 is a nuclear stain (green) that overlaps with DAPI nuclear counterstain (blue) (20X magnification) Scale bar denotes 200μM. (B) Quantitative analysis of fluorescence intensity of H3K27me3 staining relative to the number of nuclei (n=2-3, mean ± SEM, one-way ANOVA). (C) Cell Titreglo analysis of cell growth/survival of human glioma lines A172 and U251 over 3 days in culture in serum-free medium after treatment with either with vehicle control (VC), IFNγ, GSK126 and GSK126+IFNγ (n=3, mean ± SEM, two-way ANOVA). [file Image_1.jpeg]

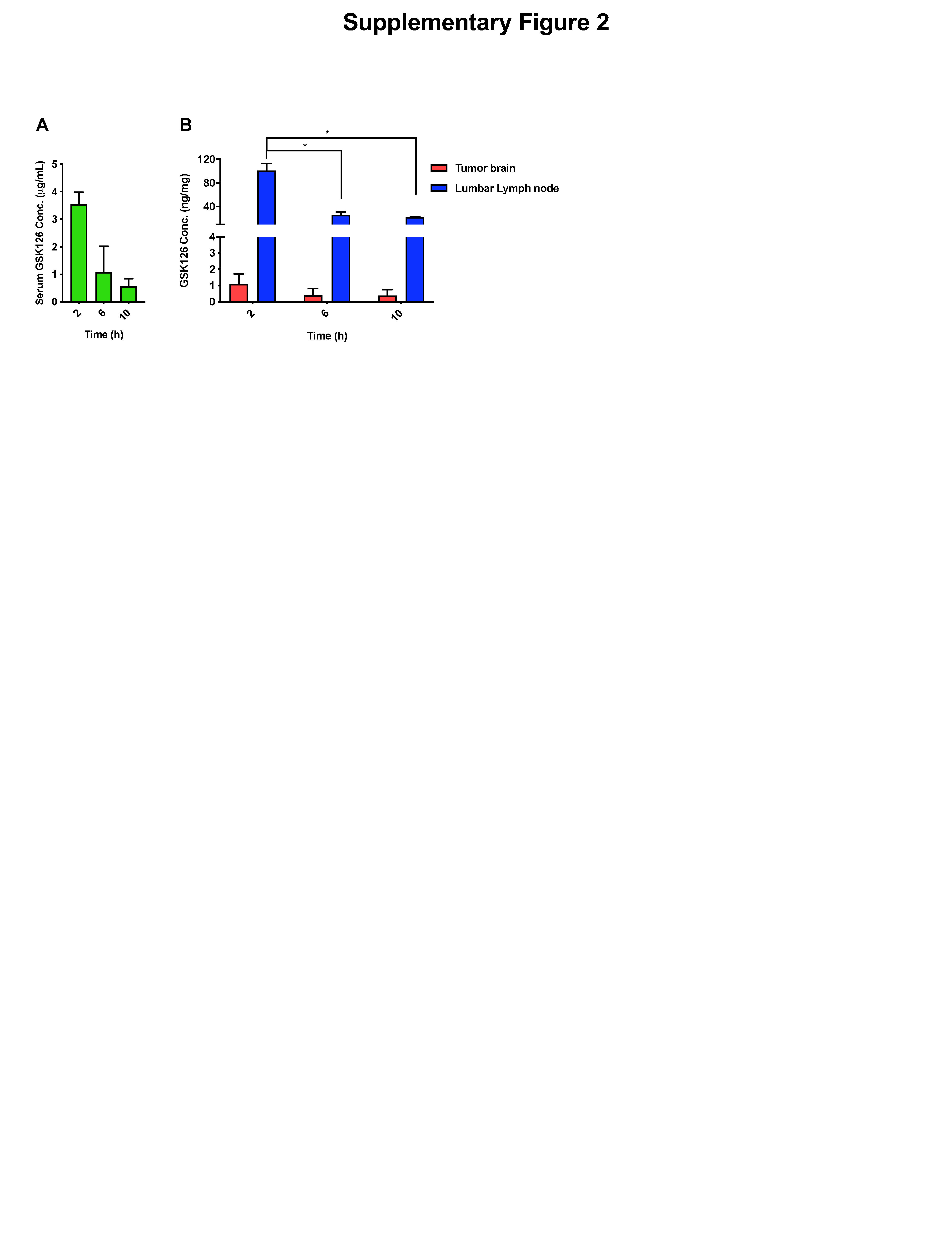

Supplement: Supplementary Figure 2 — GSK126 crosses the blood-brain barrier to reach intracranial tumors and can act both peripherally and intratumorally. (A, B) LC-MS based quantitation from serum (A), tumor tissue and lumbar lymph nodes (B) isolated from mice implanted intracranially with murine gliomas and treated with GSK126 shows that the drug can be detected above background within the tumor and peripherally (n=3 for per cohort 2h time point and n=2 per cohort for 6 and 10h time point, mean ± SEM, p<=0.05 by oneway ANOVA for A and two-way ANOVA for B). [file Image_2.jpeg]

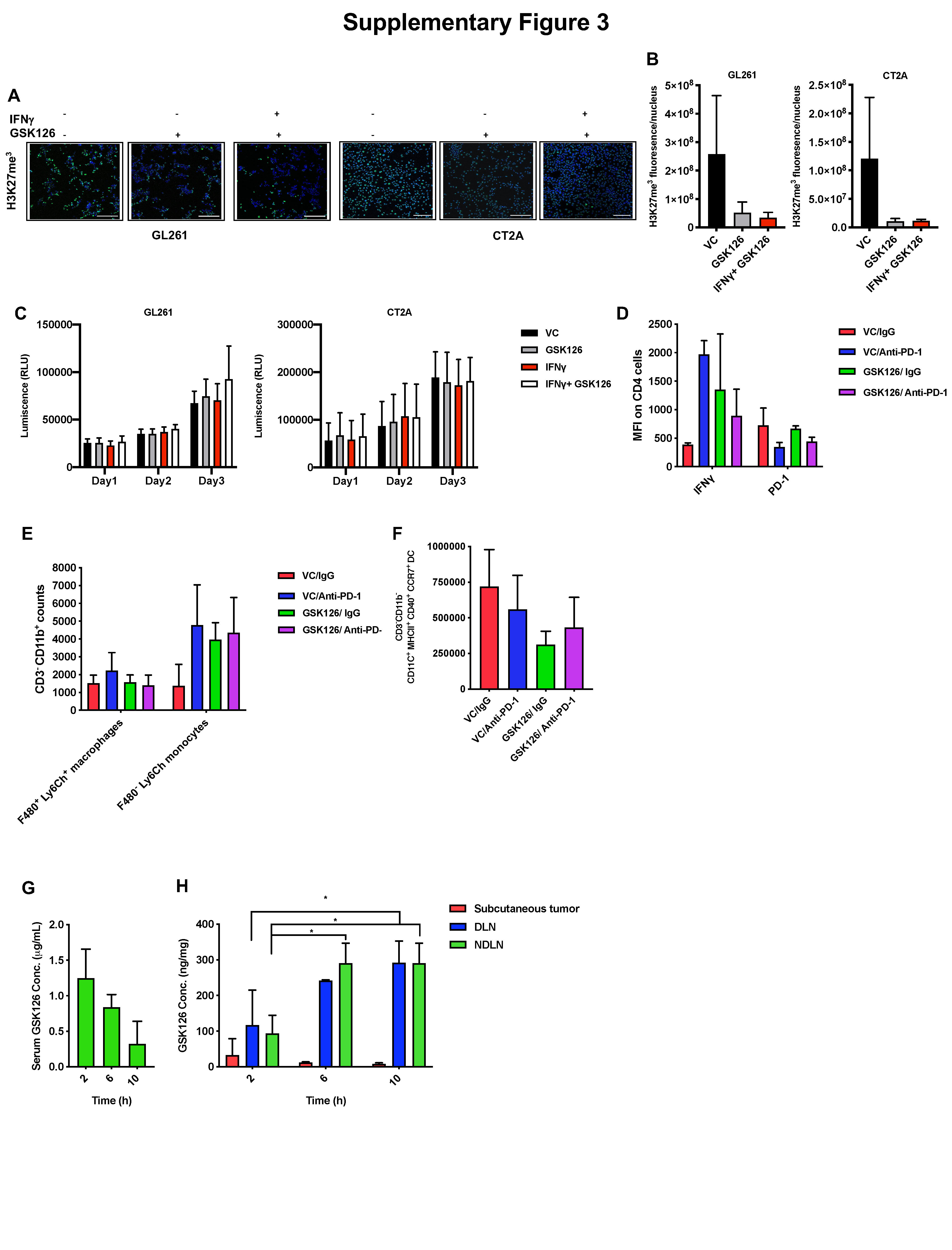

Supplement: Supplementary Figure 3 — H3k27me3 methylation is reversed with the addition of GSK126 in murine glioma cells in vitro and GSK126 can penetrate the tumor as well as lymph nodes in vivo. (A) Representative images of immunofluorescence staining of murine glioma cell lines GL261 and CT2A mCherry-ffluc stained for H3K27me3 in presence and absence of IFNγ and/or GSK126. H3K27me3 is a nuclear stain (green) that overlaps with DAPI nuclear counterstain (blue) (10X magnification). Scale bar denotes 200μm. (B) Quantitative analysis of fluorescence intensity of H3K27me3 staining relative to the number of nuclei (n=3, mean ± SEM, one-way ANOVA). (C) Cell Titre-glo analysis of cell growth/survival of murine glioma lines GL261 and CT2A mCherry ffluc over 3 days in culture in serum-free medium after treatment with either vehicle control (VC), GSK126 and/or IFNγ for 24h (n=3, mean ± SEM, two-way ANOVA). (D) Flow cytometry analysis of the median fluorescence intensity (MFI) of IFNγ and PD-1 expression on CD4+ T cells from tumor lymphocytes obtained from Figure 2E and gated from Figure 2H demonstrates that combination treatment group had no significant effect on expression of IFNγ and anti-PD-1(n=6, mean ± SEM, p<=0.05 by two-way ANOVA). (E, F) Flow cytometry analysis of myeloid cells in the draining lymph nodes obtained from mice with subcutaneous tumors in described in Figure 2E, gated from CD3- CD11b+ cells in E and CD3- CD11c+ cells in F shows no significant differences with GSK and/or anti-PD-1 treatment [n=6, mean ± SEM, two-way ANOVA (E) and one-way ANOVA (F)]. (G, H) LC-MS based quantitation from serum (G), tumor tissue and lymph nodes (H) isolated from mice implanted subcutaneously with murine gliomas and treated with GSK126 shows that the drug can be detected above background within the tumor and peripherally (n=3 for per cohort 2h time point and n=2 per cohort for 6 and 10h time point, mean ± SEM, p<=0.05 by one-way ANOVA for G and two-way ANOVA for H). [file Image_3.jpeg]

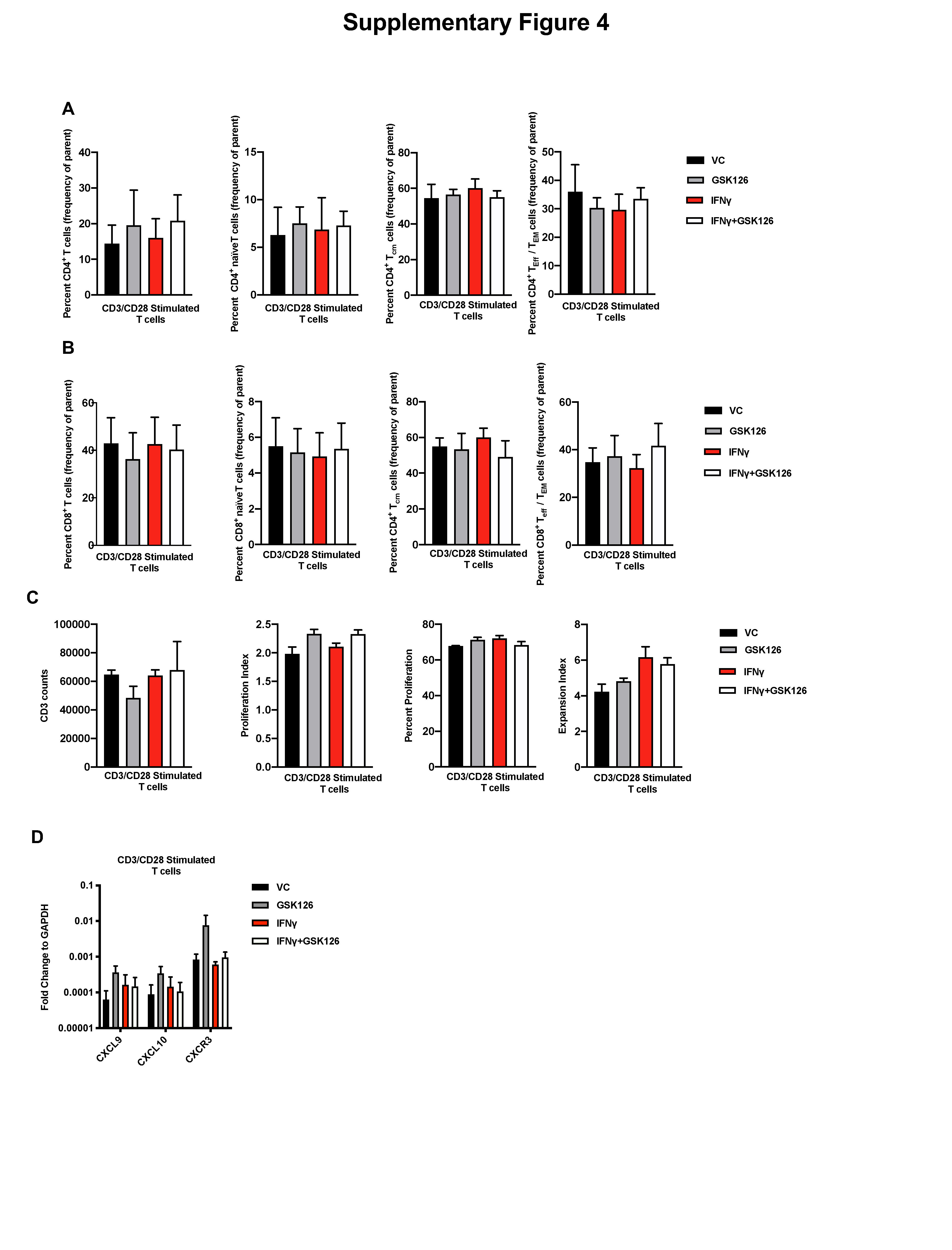

Supplement: Supplementary Figure 4 — GSK126 has no direct effect on T cell maturation and proliferation. (A, B) Flow cytometry- based analysis of T cell maturation subsets for CD4T cells (A) and CD8T cells (B) stimulated using anti-CD3/ anti-CD28 antibodies and treated with GSK126, IFNγ or the combination of the two (n=5 mice, mean ± SEM, one-way ANOVA). (C) Flow cytometry- based analysis of T cell proliferation following stimulation using anti-CD3/ anti-CD28 antibodies and treatment with GSK126, IFNγ or the combination of the two (n=2 mice, mean ± SEM, one-way ANOVA). (D) qPCR analysis of the expression of CXCL9, CXCL10 and CXCR3 in T cells following stimulation using anti-CD3/ anti-CD28 antibodies and treatment with GSK126, IFNγ or the combination of the two (n=5 mice, mean ± SEM, one-way ANOVA). [file Image_4.jpeg]
